# Efficacy of treatment with ranibizumab in patients with wet age-related macular degeneration in routine clinical care: data from the COMPASS health services research

**DOI:** 10.1007/s00417-013-2562-6

**Published:** 2014-01-15

**Authors:** Armin Wolf, Anselm Kampik

**Affiliations:** Augenklinik der LMU, Klinikum der Universität München, Campus Innenstadt, Mathildenstrasse 8, 80336 München, Germany

**Keywords:** Age-related macular degeneration, Intravitreal injections, Non-interventional study, Ranibizumab, Visual acuity, Neovascular age-related macular degeneration

## Abstract

**Background:**

To assess healthcare processes during treatment of neovascular age-related macular degeneration (AMD) in patients under real-life conditions and evaluate efficacy of monthly visual acuity (VA) assessment in a *pro re nata* treatment regime.

**Methods:**

A multicentre, prospective, non-interventional study based in Germany included neovascular AMD patients treated with intravitreal ranibizumab. Patients completed a 3-month loading phase with monthly intravitreal injections of 0.5 mg ranibizumab, followed by a 12-month maintenance phase during which investigators documented VA, additional injections, metamorphopsias, routine ophthalmological examinations and adverse events at monthly follow-up visits. Efficacy analysis included change from baseline in best-corrected VA (BCVA) based on descriptive statistics.

**Results:**

A total of 2,232 patients were enrolled throughout Germany and 1,729 patients (mean age 77.8 years, 63.2 % women) comprised the efficacy population with a complete set of data. In the clinical setting recorded in our study, only a minority of patients underwent optical coherence tomography during the maintenance phase (71 of 1,729 patients). Patients received a mean total of 4.5 injections; three injections during upload phase and 1.5 additional injections during maintenance phase. Over half of the patients (51.4 %) did not receive additional injections. Mean decimal BCVA increased during the upload phase, (from LogMAR mean of 0.201 at baseline to 0.219 at Month 4) but displayed a decline over time (0.192 at Month 15).

**Conclusion:**

Ranibizumab treatment in a real-life setting demonstrated efficacy in neovascular AMD patients, as shown by initial gains in BCVA. However, maintenance and improvement of these gains during the maintenance phase in a clinical routine setting remained below those expected compared with MARINA, ANCHOR and CATT trials, most likely due to a low number of retreatments, and the high number of patients with a poor response in regard to improvements of VA who were not investigated in these studies.

**Trial registration number:**

This phase IV non-interventional health services research study was conducted under the Novartis internal registration code, CRFB002ADE10.

## Introduction

Age-related macular degeneration (AMD) is a progressive disease and the leading cause of irreversible vision loss among people over 50 years of age in developed countries [1]. Today, approximately 20 % of people 65 to 74 years of age have findings of early macular degeneration, and at advanced age (75 to 84 years) prevalence rates of blindness in the late stage of AMD are up to 5 % [2].

In Germany, more than 370,000 people are currently diagnosed with neovascular AMD, with approximately 35,000 new cases occurring annually and calculations projecting a dramatic increase of AMD-related blindness to occur by 2030 [3, 4]. AMD can be divided into two subtypes: dry and wet (or neovascular) AMD. While the dry form is by far the most frequent, 80 % of the cases with severe vision loss in AMD are due to wet AMD [5, 6]. This is characterised by abnormal growth of new blood vessels. The age-related processes that lead to the stimulation of pathologic neovascularisation are not completely understood. However, vascular endothelial growth factor (VEGF) has been implicated as an important factor in the process of neovascularisation [7]. Blockade of VEGF has been shown to be effective in patients suffering from neovascular AMD [8, 9].

There are currently three VEGF inhibitors approved for the treatment of wet AMD in Germany: pegaptanib (Macugen*®*, Pfizer, New York City, NY, US), ranibizumab (Lucentis*®*, Novartis Pharmaceuticals, Basel, Switzerland), and recently aflibercept (Eyelea*®*, BayerPharma AG, Berlin, Germany). A fourth VEGF inhibitor, bevacizumab (Avastin*®*, Genentech, Inc., California, US/Roche, Basel, Switzerland), is prescribed ‘off-label’ for the treatment of wet AMD [10].

The PIER study [11, 12] showed that a rigid three-monthly regimen followed by ranibizumab injection every three months led to inferior treatment outcomes compared with the ANCHOR and MARINA studies using rigid monthly treatment, and PrONTO studies which used a loading phase of three consecutive monthly injections, followed by monthly monitoring and retreatment in case of recurrence [8, 9, 13]. In response to these findings, the German Ophthalmological Society (DOG), the Retinological Society and the Association of Ophthalmologists (BVA) recommended that patients in Germany should be evaluated by their treating ophthalmologist at monthly intervals and retreatment should be performed only in case of recurrence as utilised by the PrONTO studies [10]. Criteria for retreatment were adopted from the PrONTO-study [13]. Retreatment was only recommended in cases of macular thickness increase greater than 100 μm upon optical coherence tomography (OCT) examination, decrease in visual acuity (VA) of five letters or more, new haemorrhage, or new active classic choroidal neovascularisation (CNV) lesion.

The COMPASS study described here was based on these initial European dosing recommendations with a loading phase of three injections at a monthly interval followed by retreatment in case of the following: visual loss of greater than five Early Treatment Diabetic Retinopathy Study (ETDRS) letters (or Snellen equivalent), an increase of retinal thickness of more than 100 μm upon OCT assessment, a new haemorrhage, or new activity of lesion on fluorescence angiography (FA) when observed during monthly monitoring. This patient-outcome oriented variable-dosing (*pro re nata* [PRN]) regimen is applied for as long as the patient benefits [14]. The European recommendations have since been updated and currently state that ranibizumab should be administered at a dose of 0.5 mg by intravitreal injection once per month until maximum visual acuity (VA) is achieved and is stable for three monthly assessments. This is followed by maintenance therapy, based on the results of individual patient monitoring with retreatment only in case of recurrence [15].

While it is known that good results can be obtained following this PRN treatment regimen under conditions of a controlled clinical study, little is known concerning the PRN results in a clinical setting. The aim of this study was to assess and evaluate the treatment of patients with wet AMD under real-life conditions using the retreatment criteria as outlined above, as well as to evaluate the efficacy of monthly VA assessment using mean changes in best-corrected VA (BCVA).

## Materials and methods

### Patients

Patients included adult male and female patients (aged ≥18 years) with wet AMD prior to commencing treatment with ranibizumab. Wet AMD was diagnosed according to routine ophthalmologic diagnostic procedures (retinal examination by means of fundoscopy, FA, or OCT) by the investigator or the referring doctor prior to start of the documentation. Classification of images was performed by the treating physician. All treatment decisions were solely at the discretion of the investigator and the patient, according to the usual standard of care. The study sponsor did not attempt to influence the prescribing patterns of any individual investigator, and the study medication was not provided by the study sponsor.

Patient data were provided from 451 ophthalmic treatment centers in Germany. The study was approved by the appropriate ethics committee and conducted in accordance with the recommendations of the German Federal Institute for Drugs and Medical Devices on observational studies (§67 section 6 German Drug Law). Relevant national authorities were given appropriate notification of the study. Written informed consent to the collection and release of anonymous data (according to the Declaration of Helsinki) was obtained from all patients before determination of full eligibility.

### Study design

COMPASS (*Lu* *c* *entis®-Vers* *o* *rgungsforschungsstudie zur wA* *M* *D - Stellenwert der zuweisenden O* *p* *hth* *a* *lmologen im Ver* *s* *orgung* *s* *netzwerk wAMD*) was a multicentre, prospective, observational (non-interventional), 15-month study composed of a 3-month upload phase (Months 1–3) followed by a 12-month maintenance phase (Month 4–15). Patients meeting the eligibility criteria were enrolled into the three-month loading phase, where three intravitreal injections of 0.5 mg ranibizumab were administered monthly. In accordance with the ranibizumab summary of product characteristics (SmPC) valid at the time, patients were recommended follow-up visits during the maintenance phase at monthly intervals after the initial loading phase. Due to the nature of the study, visits were not scheduled solely for the purpose of data collection.

### Efficacy outcomes

Patient VA, vision loss, metamorphopsias, and results of ophthalmologic routine examinations (if available) were recorded, as well as the number of further injections administered during the 12-month maintenance phase (Month 4–15) and the reasons for not meeting the agreed visit date (if applicable). VA was assessed by means of Snellen and ETDRS charts, and was recorded decimally according to European standard EN ISO 8596 where possible. Documented VA noted as a Snellen equivalent was converted into decimal notation. In addition, finger-counting was rated as 0.01, hand movement as 0.005 and the perception of light as 0.001. Adverse events (AEs) experienced by the patient were documented by the investigator at each visit. At the final visit (Month 15), the investigator and patient were requested to rate whether monthly assessments were meaningful, and if they thought that the recommendation of monthly follow-up visits could be easily implemented from their individual perspectives.

### Statistical analysis

Efficacy analysis was performed on patients over 50 years of age with accurate and complete data (efficacy population), using a last observation carried forward imputation. Patients enrolled on the study that received ranibizumab, and for whom correct documentation and baseline data were available, comprised the safety population from which AEs were summarised using descriptive statistics. The primary endpoint of this study was the mean change in BCVA from baseline. Mean VA was calculated arithmetically and, to enhance the comparability of changes, on a logarithmic basis (a difference of 0.1 logMAR [log Minimum Angle Resolvable]-level is equivalent to 1 EDTRS-line or 5 letters). The change in BCVA over time was analysed using descriptive statistics (mean and standard error of the mean). All analyses were exploratory, no confirmatory statistical tests were performed, and no confirmatory statements derived.

## Results

### Patient population

**Fig. 1:**
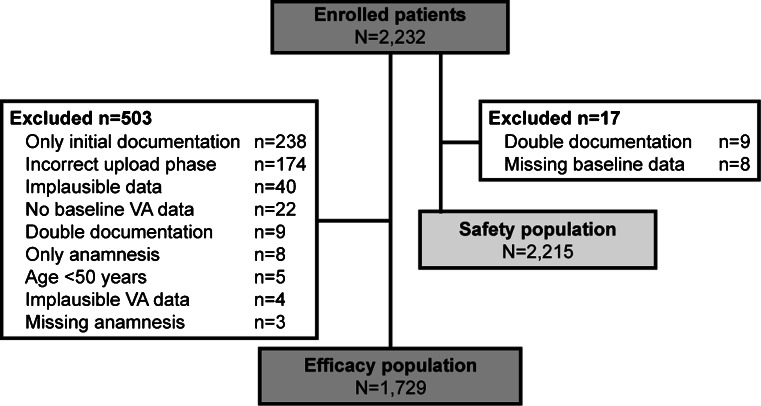
Flow diagram of patient participation. *N* number of patients; *VA* visual acuity

The first patient was screened in October 2007, and the last patient completed the study in March 2011. A total of 2,232 patients were enrolled into the study from 451 centres throughout Germany (Fig. 1). Of these 2,232 patients, 17 were excluded from analyses due to double documentation (*n* = 9) or missing baseline data (*n* = 8), thus, the safety population was comprised of 2,215 patients. A further 503 patients were excluded from the efficacy population, most commonly due to: missing maintenance phase follow-up data (*n* = 238); incorrectly performed upload phase (<3 injections or incorrect intervals between injections; *n* = 174); incorrectly recorded data (*n* = 40); and no baseline VA data gathered (*n* = 22). The efficacy population, therefore, included 1,729 patients.

Demographic and medical background data is displayed in Table 1. As expected from an AMD patient population, the mean age was 77.8 years, with half of the patients older than 78.8 years. There was an even distribution of disease between left and right eyes, with a diagnosis of wet AMD in the right eye of 865 patients and in the left eye of 841 patients. In 42.9 % (*n* = 741) of cases, both eyes showed signs of AMD. The form of CNV was occult in 41.3 % (*n* = 714) of patients, predominantly classic in 24.4 % (*n* = 422), and minimally classic in 7.8 % (*n* = 134). No data on CNV type was available for 26.5 % (*n* = 458) of patients.

**Table 1:** Demographic data and baseline characteristics of the patients (before upload-phase)

Variable (N^a^)	Efficacy population (*N* = 1,729)
Age - years (*N* = 1,718)	
Mean (SD)	77.8
Range	50.1 – 97.1
Age group – % (*N* = 1,718)	
<65 years	5.4
65-70 years	9.5
70-75 years	18.4
75-80 years	23.8
80-85 years	26.8
85-90 years	14.3
>90 years	1.7
Sex – n (%) (*N* = 1692)	
Male	599 (34.6)
Female	1.093 (63.2)
Previous treatment for AMD – n (%)^b^ ≥5 %	
None	1.171 (67.7)
Nutritional Supplements	362 (20.9)
Intravitreal injections	108 (6.2)
Risk factors - (%)^b^ ≥5 %	
Arterial hypertension	985 (57.0)
Diabetes mellitus	305 (17.6)
Dyslipidemia	199 (11.5)
Smoking	149 (8.6)
Myocardial infarction	91 (5.3)
Others	280 (16.2)
Most frequent diagnostic findings (ophthalmologic examinations) – n (%)^b,c^ ≥20 %	
Intra-/subretinal fluid	1,176 (68.0)
New lesions	953 (55.1)
Intra-/subretinal bleeding	692 (40.0)
Best-corrected VA (*N* = 1,729)	
Arithmetic mean (SD)	0.277 (0.190)
Logarithmic mean	0.201
Intraocular pressure - mean (SD) (*N* = 1,538)	15.22 (2.92)

*AMD* age-related macular degeneration; *SD* standard deviation

^a^Patients with available data; ^b^multiple naming possible; ^c^residual patients were stated as “no” or “not specified”

### Change in BCVA

**Fig. 2:**
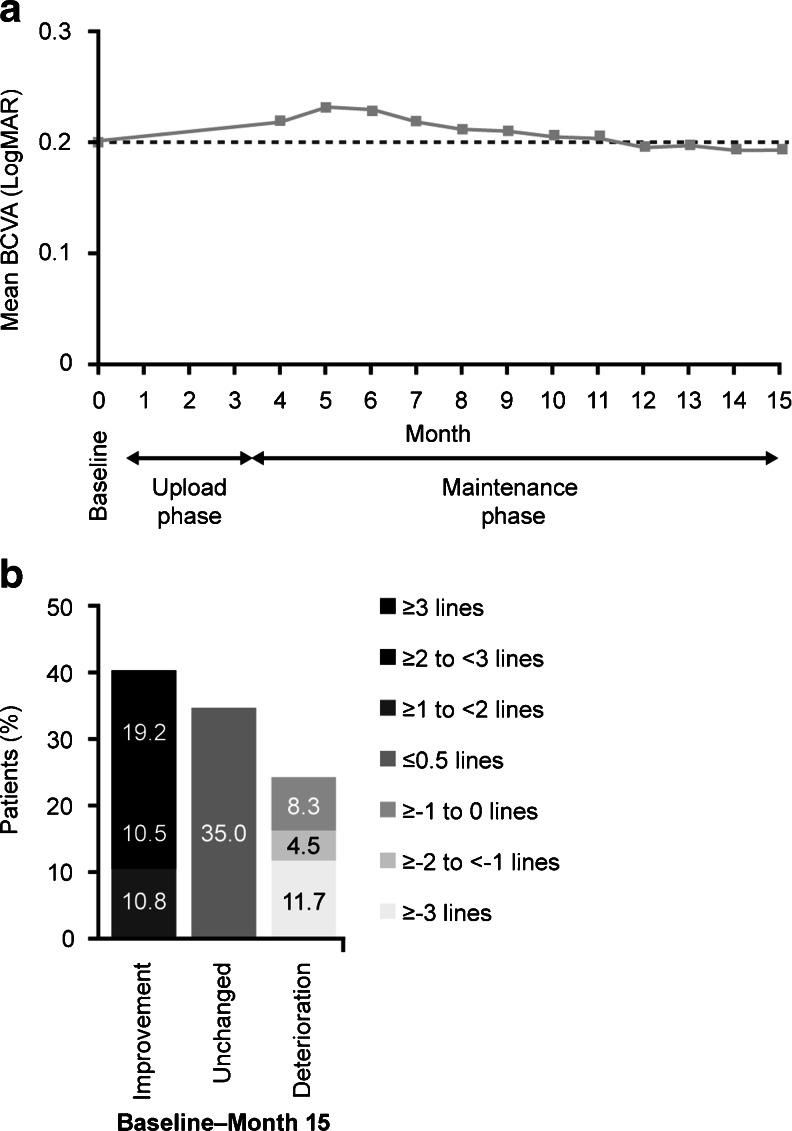
Change in BCVA during the study: **a** Mean BCVA during the study (Efficacy population, LOCF) according logMAR values. **b** The change in BCVA from baseline–Month 15 and Month 4–Month 15 according to the percentage of patients who improved (>0 lines), remained unchanged, or declined (<0 lines), by logMAR BCVA. *BCVA* best-corrected visual acuity; *LOCF* last observed carried forward

Mean BCVA increased during the three-month upload phase, from a LogMAR mean of 0.201 at baseline to 0.219 at Month 4 (Fig. 2a). Improvement in BCVA from baseline was maintained up to the second follow-up visit during the maintenance phase, at Month 5. Subsequent to this visit, mean BCVA declined steadily over time during the maintenance phase, from 0.233 at Month 5 to 0.192 at Month 15 (Fig. 2a). During the study (Month 0–15), improvement in BCVA was displayed by 40.5 % of patients, deterioration by 24.5 % of patients and no change by 35.0 % of patients (Fig. 2b).

### Self-assessment of VA

Changes in VA as measured by the investigator were in accordance with patient self-assessment records (data not shown). At the first follow-up visit during the maintenance phase (Month 4), patients were asked if they had experienced any vision loss. At each subsequent visit (Months 5–15), patients were asked to classify their vision as ‘better’, ‘unchanged’ or ‘worse’ as compared with the previous visit. Following the three ranibizumab injections administered during the upload phase, 29.8 % of patients evaluated their VA as ‘better’ at Month 5. Thereafter, the percentage of patients who evaluated their VA as ‘better’ declined to 14.7 % at Month 15. The percentage of patients who did not perceive any change in VA during the maintenance phase increased from 57.8 % at Month 5 to 73.4 % at Month 15, compared with the percentage of patients who evaluated their vision as ‘worse’, which remained similar throughout the study (12.3 % at Month 5 versus 13.8 % at Month 15). Patients were also asked to evaluate metamorphopsia; the majority of patients classified metamorphopsia as ‘unchanged’ throughout the study (55.8 % at Month 5; 70.1 % at Month 15).

### Retreatment frequency during maintenance phase

Following the three initial monthly injections during the upload phase, 840 (48.6 %) patients received additional injections during the 12-month maintenance phase (average of 3.1 injections per patient, range 1 to 11 injections). In the efficacy population (*n* = 1,729), an average of 1.5 additional injections were administered per patient during the maintenance phase, resulting in an average of 4.5 ranibizumab injections received per patient in total (three injections during upload phase plus 1.5 injections during maintenance phase). The percentage of patients receiving additional injections increased steadily during the maintenance phase from Month 4 (14.2 %) to Month 8 (19.1 %), then subsequently decreased each month until the end of the study (13.6 % at Month 15).

### Subgroup analysis

**Fig. 3:**
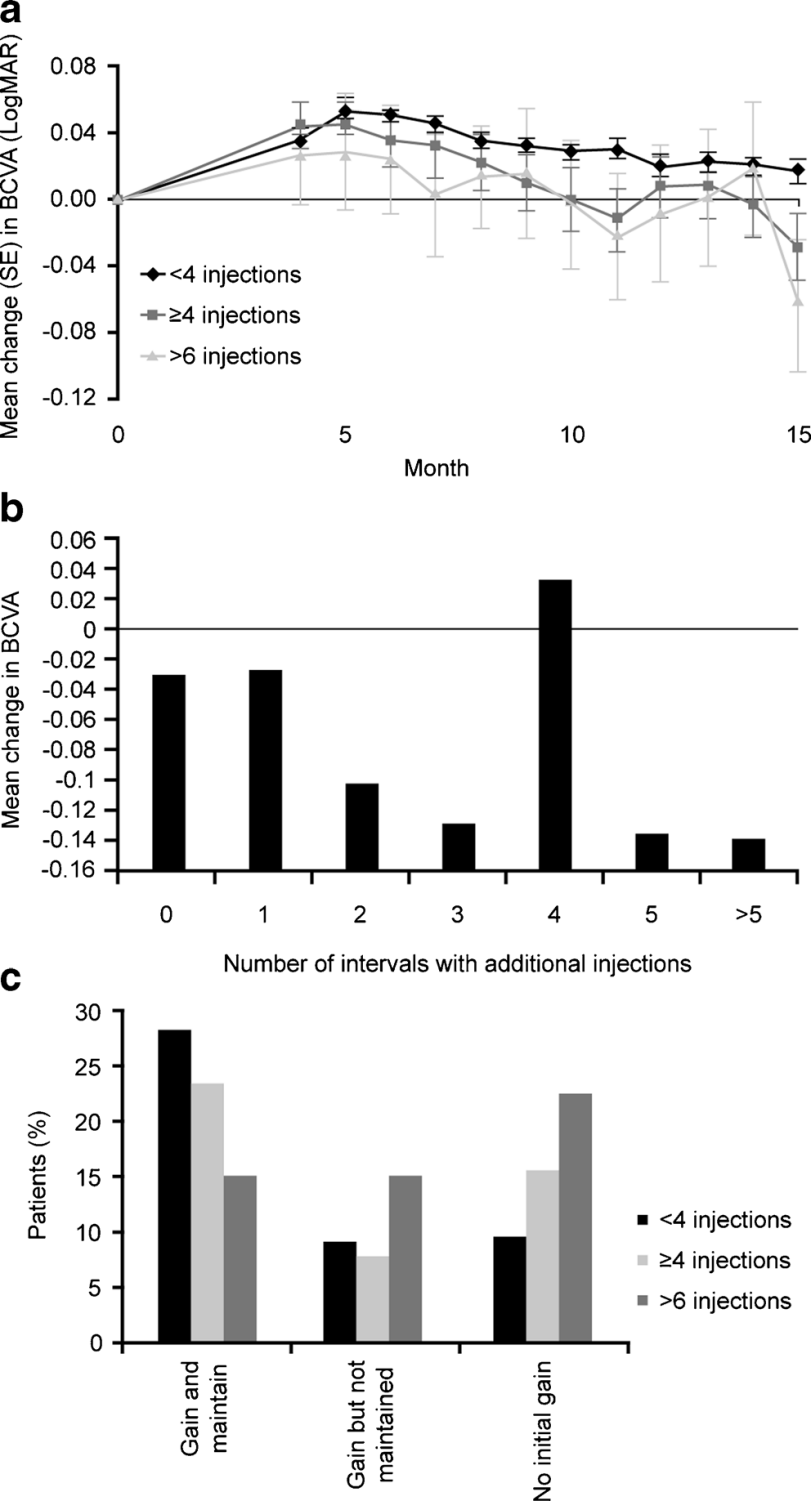
Change of VA from baseline to Month 15 according to the number of additional injections: **a**, **b** Mean change in BCVA from baseline-Month 15 according to the number of additional injections received during the maintenance phase. **c** Percentage of patients receiving additional injections according to criteria ‘Gain and maintain’ (BCVA at baseline < BCVA at Month 4 < BCVA at Month 15), ‘Gain but not maintain’ (BCVA at baseline < BCVA at Month 15 < BCVA at Month 4), and ‘No initial gain’ (BCVA at baseline > BCVA at Month 4 < BCVA at Month 15)

Subgroup analysis according to the number of retreatments received during the maintenance phase demonstrated that all patients who received additional injections during the maintenance phase presented a mean BCVA at Month 15 which was below that achieved after the upload phase at Month 4 (Month 15: <4 injections, *n* = 1,045; ≥4 injections, *n* = 136; >6 injections, *n* = 37, Fig. 3a). However, patients who received fewer than four additional injections of ranibizumab during the maintenance phase displayed less decline from baseline in BCVA over the course of the study and levels did not fall below baseline, compared with patients receiving more than four injections (Fig. 3a). Analysis of the change in BCVA from baseline to Month 15 according to the number of additional injections received by the patient during the maintenance phase (0 injections, *n* = 889; 1 injection, *n* = 186; 2 injections, *n* = 195; 3 injections, *n* = 240; 4 injections, *n* = 85; 5 injections, *n* = 68; >5 injections, *n* = 66) shows that patients who received one additional injection and those who did not receive any additional injections both maintained similar levels of BCVA during this time (both displayed a change from baseline to Month 15 of -0.002 LogMAR; Fig. 3b). In comparison, patients receiving two, three and five or more additional injections experienced a loss in BCVA greater than 0.03 LogMAR during the maintenance phase (Fig. 3b). Only those patients who received additional injections at four follow-up visits during the maintenance phase showed an improvement in BCVA (+0.016 versus baseline; Fig. 3b).

Change in BCVA during the study for patients receiving additional injections was also analysed according to the following responder group definitions (Fig. 3c): ‘Gain and maintain’ (BCVA increases from baseline to Month 4 and then to Month 15), ‘Gain but not maintain’ (BCVA increases from baseline to Month 15, but with a decline from Month 4 to end of study), and ‘No initial gain’ (BCVA declines from baseline to Month 4, but increases from Month 4 to Month 15). Most of the patients classified as ‘Gain and maintain’ received fewer than four additional injections during the maintenance phase (28.3 %), whereas the majority of patients classified as ‘Gain but not maintain’ and ‘No initial gain’ received more than six additional injections (15 % and 22.5 %, respectively) (Fig. 3c). During the maintenance phase, patients showed profit from therapy as documented by the increase of VA at the visit following the injection.

### Feasibility evaluation by investigators and patients

**Fig. 4:**
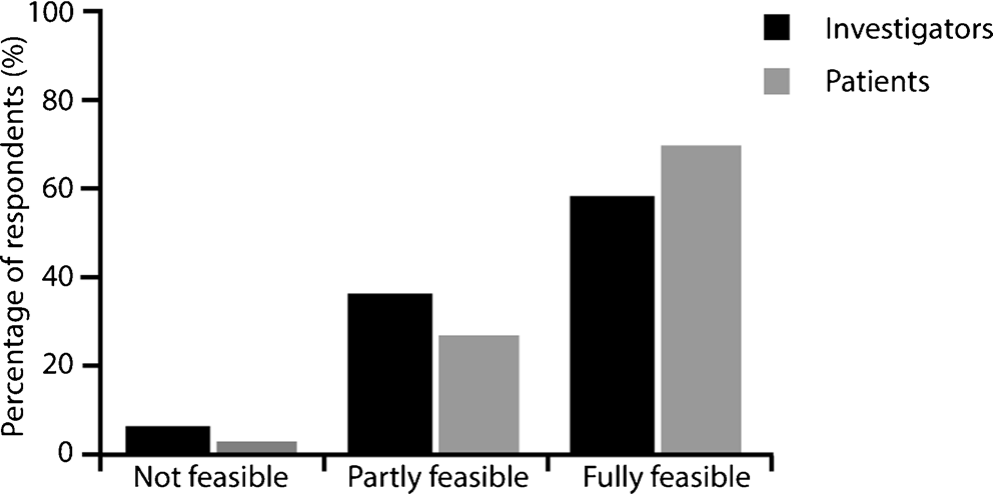
Feasibility of the SmPC recommended monthly follow-up visits in practice, as perceived by investigators and patients. *SmPC* summary of product characteristics

Investigators and patients were requested to rate whether monthly assessment was meaningful and if the monitoring recommendations provided in the SmPC could be easily followed from the investigator’s and the patient’s perspectives (Fig. 4). Of the 288 investigators and 1,369 patients who responded, 58.0 % of the investigators and 70.3 % of the patients found implementation of the recommended monthly assessment into routine care to be fully feasible. Amongst those investigators who found monthly assessment partly feasible (36.5 %), the most common reasons given were due to: cost, logistics and patient number (*n* = 89); patient age and multi-morbidity (*n* = 18); and compliance problems (*n* = 16). For investigators who found monthly assessment not feasible (5.6 %), reasons given were due to: cost, logistics and patient number (*n* = 9), and lack of reimbursement (*n* = 9). The most common reasons given by patients who found monthly assessment partly feasible (26.9 %) were: too high burden (*n* = 82); transportation issues (*n* = 76); and the high time demands (*n* = 58). Patients who found monthly assessment not feasible (2.8 %) most commonly gave the reasons: too high burden (*n* = 15); transportation issues (*n* = 5); and the high expenditure of time (*n* = 5).

### Tolerability

The safety analysis was based on data from 2,215 patients (Table 2). Investigators reported a total of 364 AEs in 123 patients during the entire observation period (AE incidence rate 5.6 %). Of those, 255 AEs in 118 patients were serious AEs (SAEs; incidence rate 5.3 %). For the majority of SAEs (*n* = 171; 67.1 %), any relationship to ranibizumab treatment was not suspected. In 14.5 % of cases (*n* = 37), a relationship to the treatment was suspected (unknown or no information for 47 SAEs). A total of 25 (1.1 %) patients discontinued the study due to an AE.

**Table 2:** Adverse events after 12 months of observation

	Safety population (*N* = 2,215) n (%)
Total (AE, SAE and SE)	364 (16.4)
SAE	171 (67.1)
SSE	84 (32.9)
Recovered	85 (33.3)
Unchanged	58 (22.7)
Deteriorated	8 (3.1)
Death	37 (14.5)
Unknown/not reported	67 (26.3)
Most frequently (*n* ≥3) reported AE (SOC-term)	
Eye disorders	18 (0.81)
Cardiac disorders	5 (0.23)
Injury, poisoning, complications	3 (0.14)
Most frequently (*n* ≥10) reported SAE (SOC-term)	
Eye disorders	46 (2.08)
Surgical and medical procedures	23 (1.04)
Injury, poisoning, complications	17 (0.77)
General disorders and administration site conditions	16 (0.72)
Cardiac disorders	15 (0.68)
Neoplasms benign, malignant and unspecified	13 (0.59)

*AE* adverse event; *MedDRA* Medical Dictionary for Regulatory Activities; *SAE* serious adverse event; *SE* side effect; *SOC* system-organ-class (according to MedDRA); *SSE* serious side effect

## Discussion

The present non-interventional study in 1,729 patients observed the pharmacological treatment with ranibizumab of wet AMD under routine clinical conditions. Patients received a mean total of 4.5 injections during the 15-month study, three during the upload phase and a further 1.5 injections on average during the maintenance phase. Although most patients presented an improvement in or a stabilisation of VA, the overall treatment efficacy remains below that expected from previous controlled clinical trials.

In previous randomised trials the number of injections received by patients was considerably higher: in the CATT study, patients were treated with a mean of seven additional injections during the first year [16]. In the ANCHOR study, the mean number of ranibizumab injections administered during two years of treatment was 21.3, and in the SUSTAIN study patients received an additional 2.7 injections during the 8-month maintenance phase [9, 17].

Data from a phase IV uncontrolled, non-interventional trial cannot be compared with the results of prospective controlled phase II or III studies with accurate VA testing and motivated patients. However, the striking difference in retreatment shows that treatment in a clinical setting daily routine may differ from that applied in controlled clinical trials. Real-world data outside of a clinical trial setting suggest that a considerably lower number of patients received the number of injections required to maintain or improve VA during the maintenance phase using the previously specified retreatment criteria. It could be argued that, although patients are being examined on a regular basis, recurrence of the disease is treated too late. This may be due to the fact that the criteria for retreatment adopted from the PrONTO study are too broad to effectively treat a recurrence under a routine clinical setting. Several studies have shown that maintenance of VA is possible based upon a PRN regimen [13, 16–18]. In these studies, a composite criterion was applied to assess the need for repeated injections (functional vision loss and morphologic changes of the macula and retina by OCT). Data from uncontrolled, retrospective analyses show that a PRN regimen leading to fewer than five injections in the first year are insufficient to sustain VA improvements [19, 20]. However, this treatment strategy was long regarded as sufficient and was based on a mathematical drug and disease model (involving data from the MARINA, ANCHOR and PIER studies) estimating 8.1 ranibizumab injections to be needed during the first year [21]. In a real-life setting, it seems that these retreatment criteria are not sufficient to adequately detect recurrence, as during the maintenance phase, VA only partly recovers to the same level achieved after upload therapy when measured by ETDRS charts [22].

One reason for the comparably low overall development of VA in this study might, therefore, be the fact that most of the patients received clinical examination using decimal VA testing. As retreatment is based upon a five-letter decrease of VA, the standard VA testing using decimal VA charts might not be sensitive enough to detect recurrence early enough [23]. Additionally, clinical studies are usually based upon VA testing using the ETDRS charts allowing a more sensitive assessment of the development of VA. Usually, the change in VA in clinical trials is reported in letters while the VA increase in this study is primary recorded using the Snellen VA testing.

Another reason, which may contribute to the overall late detection of recurrences, might be the fact that only a small number of patients (*n* = 71) received OCT examinations during maintenance phase assessments. It has been shown that changes indicating a recurrence of wet AMD can be seen upon close spectral domain (SD)-OCT examination before deterioration of VA [13, 18, 21, 24] and recent studies such as CATT are based upon OCT retreatment-criteria. Following this, the ophthalmologic societies (DOG, BVA and the German Retina Society [RG]) recently recommended implementation of morphologic criteria for re-injection (i.e., new haemorrhage, findings from morphologic criteria on OCT) which are more sensitive to changes in the retina than functional criteria [10]. However, at the time of the study, these retreatment criteria were not present, thus, only a minority of the patients were examined by OCT.

The authors consider it noteworthy that patients receiving four injections of ranibizumab during maintenance displayed the best visual outcomes. With a maximum of three additional treatments during upload phase, these patients received up to seven injections during a follow-up period of 15 months, which is in accordance with the findings from the CATT study (6.9 injections during a 12-month follow-up period) [16]. Interestingly, patients receiving more than five additional injections did not present a better improvement of VA at 15 months, compared with those who received four additional injections. Although one should be cautious in interpreting data from an uncontrolled non-interventional study, this could be due to the fact that these patients represent poor responders to anti-VEGF treatment in regards to improvement of VA. However, additional pathology, such as the presence of geographic atrophy or fibrosis, may also be responsible for limiting the response to anti-VEGF treatment. It seems striking that under real-life conditions, the proportion of poor responders seems higher than in clinical studies (e.g., SUSTAIN study) [17]. However, to our understanding this can be explained by strict inclusion criteria in these studies, which were not present in our study. Additionally, in a routine clinical setting there seems to be a high number of patients with a lack of VA increase during upload therapy, and these patients are less motivated to undergo the burden and the logistic problems of an additional injection. Interestingly, most patients and investigators found monthly assessment at least partly feasible. Of note, the most prominent reasons for refusal of monthly assessments were logistical problems and high costs for all parties.

In the study described here, although initial gain of VA after upload treatment could not be maintained during maintenance, VA at 12 months was stable compared with VA at baseline. This stabilisation of VA was also found in the overall self-assessment of the patients: 73 % of patients stated that they did not perceive any change in VA compared with baseline.

Our results are in accordance with a recent study by Heimes and colleagues reporting that the flexible, predominantly VA-driven ranibizumab retreatment regimen employed in clinical practice in Germany, generally results in a loss of BCVA during the 12 months of follow-up following initial gains during upload phase [25].

Lastly, a lower than expected number of reported AEs was observed during this study, with only a 5.6 % incidence rate. The majority of these AEs were classed as SAEs (255 of 364 AEs, 5.3 % incidence rate). In comparison, CATT reported an SAE incidence rate of 31.7 % in the ranibizumab treatment arm over two years [16]. These findings suggest that there is a lack of reporting of both AEs and SAEs in a real-world setting, as opposed to in a formal clinical trial.

## Conclusion

This study showed that ranibizumab is effective in improving VA in patients with wet AMD, as shown by the initial positive treatment effects and short-term effects after retreatment. Although most patients presented either with VA gain or stabilisation during the 15 months follow-up, the overall treatment success was lower than that observed in controlled clinical trials. This indicates that the former retreatment criteria derived from the PrONTO study are not sufficient to maintain VA during maintenance phase of anti-VEGF treatment under real-life settings. Considering the low retreatment rate when compared with controlled clinical trials, it seems that in the clinical routine setting recurrences are detected later than in clinical trials. Thus, new retreatment criteria with regular assessments of vision (i.e., VA assessments by ETDRS charts), or detection of macular oedema increase by SD-OCT should be employed in a clinical routine setting, as recommended by the DOG and BVA and by the amended SmPC [26].
